# Neurosonographic Evaluation of Cerebral Cortical Development in Fetuses With Congenital Heart Disease: A Systematic Review of the Literature

**DOI:** 10.1002/pd.6862

**Published:** 2025-07-25

**Authors:** Marcelo Dantas Cerqueira Monteiro, Thatiane Lopes Valentim Di Paschoale Ostholin, Miriam Pérez‐Cruz, Luana Sarmento Neves da Rocha

**Affiliations:** ^1^ Fetal Medicine Caliper Escola de Imagem Salvador Brazil; ^2^ Departamento de Ciências do Movimento Humano Universidade Federal de São Paulo São Paulo Brazil; ^3^ BCNatal/Fetal Medicine Research Center (Hospital Clínic and Hospital Sant Joan de Déu) University of Barcelona Barcelona Spain; ^4^ Institut de Recerca Sant Joan de Déu Barcelona Spain; ^5^ Department of Obstetrics and Ginecology Fetal Medicine Universidade Federal da Bahia Salvador Brazil

## Abstract

This systematic review collated data from neurosonography and ultrasound evaluations to assess changes in the cortical development of fetuses with congenital heart disease (CHD). Of the 135 articles identified by two independent reviewers, five that satisfied our inclusion criteria were selected. Cortical evaluation was performed by 2D and/or 3D ultrasound via either a transabdominal or transabdominal plus transvaginal approach. One study used a brain‐age evaluation algorithm, while the other four measured the depths of brain fissures. Gestational age at the time of fetal evaluation was very heterogeneous, from 20 to 37 weeks. Four of the studies included several types of CHD but one included only fetuses with tetralogy of Fallot. All five studies detected significant delays in the development of brain sulci and gyri in CHD fetuses compared to controls. In general, the studies were of good methodological quality, but all showed some risk of bias. The main methodological issue was the lack of comparison of ultrasound findings with magnetic resonance imaging data. To conclude, ultrasound was found useful in the evaluation of fetal cortical development, showing that fetuses with CHD present some degree of delayed cortical development, but postnatal studies are needed to understand if it correlates with impaired neurodevelopment.

## Summary

- What is already known about this topic?
  ◦ Congenital heart diseases are associated with impaired neurodevelopment in children and adolescents. Studies suggest that signs of abnormal neurological development appear prior to cardiac surgery. Research using magnetic resonance imaging has shown signs of delayed brain development in fetuses with congenital heart defects.
- What does this review add?
  ◦ The evidence provided by this comprehensive review suggests that cortical development delays in fetuses with isolated cardiac abnormalities exist and that ultrasound can detect such delays.

## 1 Introduction

Congenital heart diseases (CHD) occur in 0.7% of live human births and are the most common type of congenital anomaly. They are also among the major causes of neonatal morbidity and mortality [1]. CHD are associated with impaired neurodevelopment in children and adolescents, even in the absence of genetic syndromes [2]. Advances in prenatal diagnostics, surgical techniques, and medical treatments have led to improved long‐term survival in this population, which has shifted the research focus from cardiac morbidity and mortality toward the neurodevelopmental outcomes of these patients [3, 4, 5, 6].

Traditionally, neurodevelopmental delays in people born with CHD have been attributed to perioperative hypoxia or thromboembolic events during surgery and long periods of hospitalization in intensive care units. However, studies suggest that signs of abnormal neurological development may be present prior to surgery [6, 7, 8].

Research using magnetic resonance imaging (MRI) has shown signs of delayed brain development in fetuses with CHD [9, 10] and these findings have been confirmed by recent neurosonographic studies [11, 12, 13]. However, the methods of evaluation in the latter were heterogeneous, with different biometric measurements [11, 12] or subjective assessments of different brain sulci and gyri [13].

Neurosonography is the method of choice when fetal central nervous system (CNS) malformations are suspected, with MRI used only as a secondary approach, with precise indications [14]. Since neurosonography with standardized measurements allows deep evaluation of the fetal cortex using direct and indirect signs, it is reasonable to assume that it can be used to assess the cortical development of fetuses with CHD.

### 1.1 Hypothesis and Objectives

We hypothesized that fetuses with isolated cardiac defects have some degree of cortical development delay compared with control fetuses and that such delays can be evaluated by targeted neurosonography or specialized ultrasound.

The main objective of this systematic review was to collate and evaluate evidence of changes in cortical development in CHD fetuses obtained by targeted neurosonography or ultrasound. The heterogeneity of the methods used to evaluate cortical development in CHD fetuses among the different studies was also object of investigation.

## 2 Methods

### 2.1 Study Design and Protocol

This systematic review was developed, conducted, and reported according to the recommendations of the Preferred Reporting Items for Systematic reviews and Meta‐Analyses (PRISMA) and Synthesizing Evidence from Diagnostic Accuracy Tests (SEDATE) guidelines [15, 16]. The scope of the study was defined using an adaptation of the Population, Intervention, Comparison, Outcome (PICO) method and the Population, Index test, Target condition (PIT) method (Table 1).

**TABLE 1:** Scope of the study according to the adapted Population, Index test, Target condition (PIT) method.

Population	Fetuses with isolated congenital cardiac disease that did not undergo prenatal cardiac surgery
Index test	Neurosonography or ultrasound
Target condition	Changes and/or abnormalities in cortical development

### 2.2 Search Strategy

PIT was used to develop our search criteria. These were fetuses with CHD and the use of neurosonography or ultrasound in cortical development evaluation. Relevant health science descriptors/medical subject headings (DeCS/MeSH) terms were then identified [17]. Additional terms were identified using PubReMiner [18]. We made minimal adjustments to the search strategy to better suit the databases of interest. The search was conducted between January 22 and 24, 2024. It was further refined after exploratory surveys. Details of the search strategy are provided in Appendix A.

### 2.3 Information Sources

Systematic searches were performed in PubMed, Epistemonikos, Biblioteca Virtual em Saúde, and the Cochrane Library. The gray literature was not searched. The reviewers used the references of the studies found to identify other relevant works.

### 2.4 Eligibility

The study inclusion criteria were (1) cross‐sectional and prospective observational studies that (2) reported the evaluation of cortical development (3) using neurosonography or ultrasound (4) in at least three fetuses (5) with CHD. We excluded case reports, studies with incomplete methodological information, studies that used only MRI evaluations, studies that only evaluated children postnatally, and those including fetuses with genetic or structural abnormalities other than the cardiac malformations or fetuses that underwent prenatal cardiac surgery. Secondary studies, editorials, opinion articles, letters to the editor, commentaries, and abstracts in Congress annals were considered ineligible. There were no restrictions on the language or the date of publication.

### 2.5 Studies Selection

Studies identified were saved in ris or csv format and uploaded to Rayyan software [19]. Two independent reviewers (M.M. and L.S.) first reviewed the titles and abstracts and excluded non‐relevant search results. The eligibility of the remaining results was then verified through retrieval and reading of the full text. Any discordance between the reviewer's selections was resolved by discussion.

### 2.6 Data Extraction and Synthesis

Data were extracted independently by two reviewers (M.M. and L.S.) using an EXCEL spreadsheet (Microsoft Corporations, Redmond, WA, USA). The following data were extracted and recorded on a datasheet: authors; date of publication; study setting; study design; study date range; participant characteristics (in each group when more than one); inclusion and exclusion criteria; sample size; method used to establish gestational age (GA); ultrasound examination characteristics, such as fetuses' GA at the time of each exam, exam type (2D and/or 3D), and approach used (transvaginal and/or transabdominal); method used to evaluate cortical development and report the results; examiner and researcher expertise levels; blinding of examiners and researchers to fetus group; postnatal evaluation of cortical development; and postnatal evaluation of long‐term neurodevelopment.

### 2.7 Assessment of Methodological Quality and Risk of Bias

Assessment of the methodological quality of the studies was performed independently by two reviewers (M.M. and L.S.) using the Newcastle‐Ottawa Scale (NOS) tool [20], which classifies the studies as of good, fair, or poor quality depending on the evaluation in three domains: selection, comparability and outcome. The risk of bias and applicability of the studies were evaluated independently by the same reviewers using the Quality Assessment of Studies of Diagnostic Accuracy Included in Systematic Reviews (QUADAS‐2) tool [21], which evaluates four key domains: patient selection, index test, reference standard, and flow and timing. Each domain is assessed in terms of risk of bias, and the first three domains are also assessed by concerns regarding applicability. These categorize the studies as having low, high, or unclear risk of bias and/or concerns regarding applicability according to the reviewers' judgement of each domain.

## 3 Results

### 3.1 Study Selection

**FIGURE 1:**
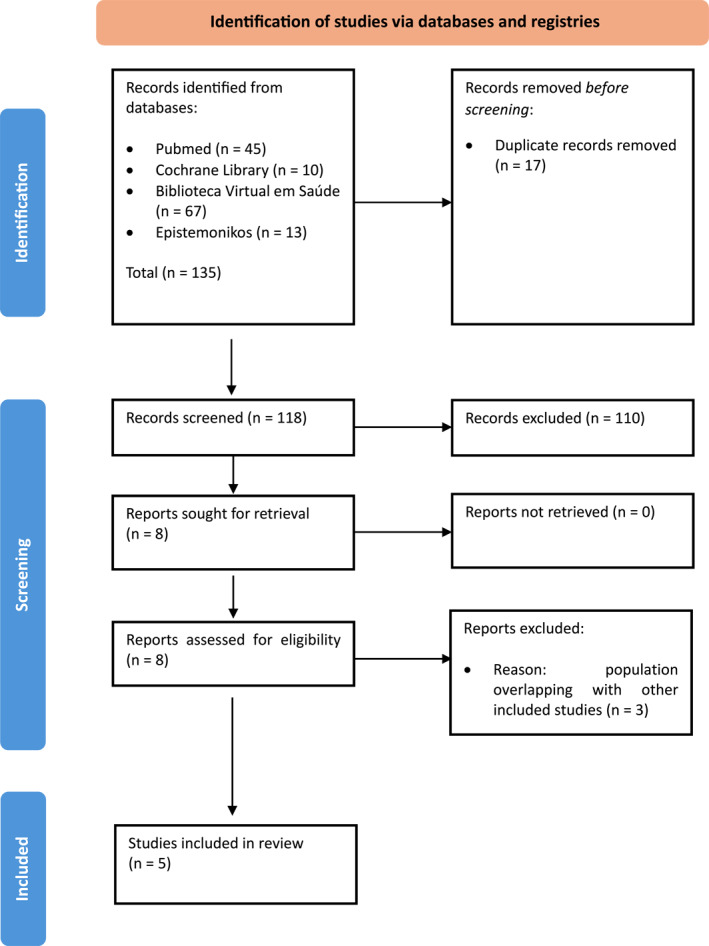
Flowchart of the studies included.

A total of 135 articles were identified through our database searches. Of these, 17 were duplicates and therefore were excluded. Another 110 were excluded after reviewing the title or abstract for one of the following reasons: inadequate publication type (61), inadequate population (31), diagnostic methods other than ultrasound or neurosonography (13), non‐relevant outcomes assessed (3), and inadequate study design (2). The full manuscripts of the remaining eight articles were retrieved. Of these, three were excluded due to superposition of the population with those from other studies. Finally, five articles [11, 12, 13, 22, 23] fulfilled the inclusion criteria and were included in our review. The study selection process is depicted in a flowchart in Figure 1.

### 3.2 Study Characteristics

The characteristics of the studies reviewed are summarized in Table 2. Four of the five studies were prospective observational studies [11, 12, 13, 22] and one was cross‐sectional [23]. One study evaluated fetal cortical development using an automated brain‐age algorithm [13], while the other four took quantitative measurements of brain fissure depths [11, 12, 22, 23]. The GA at assessment was very heterogeneous and varied from 20 to 37 weeks. Four studies included different types of CHD [11, 12, 13, 23] and one included only fetuses with ToF [22]. All five studies detected significant delays in brain sulci and gyri development of the CHD fetuses compared to controls. None of the included studies evaluated postnatal cortical development, either by ultrasound or MRI, and only one evaluated postnatal neurodevelopment in the medium or long term for comparison with prenatal findings [11].

**TABLE 2:** Detailed characteristics of the studies included.

Author	Study design	Country of study	Cases (*N*)	Controls (*N*)	US type and approach	Type of measurement to evaluate cortical develop.	GA of evaluat.	Criteria used to interpret evaluat.	Subgroups of cardiac diseases	Postnatal cortical evaluat.	Medium and/or long‐term neurodev. evaluat.
Peng et al. (2016)	Prospective cohort	China	45	45	2D, abdominal	SF depth^a^, POF depth^b^, CF depth^c^	20–25 weeks, 26–31 weeks and 32–37 weeks	Comparison of brain fissure depths between cases and controls	Three subgroups: HLHS RHOL TGA	No	SFs positively correlated with PDI (*r* = 0.608, *p* < 0.01) and MDI score (*r* = 0.435, *p* < 0.01)
Koning et al. (2017)	Prospective cohort	The Netherlands	20	193	3D, abdominal and transvaginal	Insula depth, SF depth and POF depth	22, 26 and 32 weeks	Differences in the trajectory of brain fissure depths between cases and controls	No subgroups	No	No
Everwijn et al. (2020)	Prospective cohort	The Netherlands	142	75	3D, abdominal	Automated algorithm based on brain predicted GA	Every 4 weeks between 20 and 33 6/7 weeks	Differences in brain GA and true GA using the algorithm	Six subgroups: Low, mixed or normal oxygen delivery to the brain; or reversed, obstructed or normal aortic arch flow	No	No
He et al. (2017)	Prospective cohort	China	40	196	2D, abdominal	SF depth^a^, POF depth^b^, CF depth^c^	23–24, 25–26, 27–28, 31–33 weeks	Comparison of brain fissure depths between cases and controls	Only fetuses with ToF	No	No
Rizzo et al. (2022)	Prospective cross‐sectional	Italy	78	80	2D, abdominal and transvaginal	Insula depth^a^, FS depth^a^, POF depth^b^, CF^c^	Single evaluation between 30 and 33 week	Comparison of brain fissure depths between groups	Group A: Mildly reduced or normal oxygenation Group B: Moderately to severely reduced oxygenation	No	No

Abbreviations: CF, calcarine fissure; evaluat., evaluation; GA, gestational age; HLHS, hypoplastic left heart syndrome; MDI, Mental Development Index; *N*, number of cases; neurodevelop., neurodevelopment; PDI, Psychomotor Development Index; POF, parieto‐occipital fissure; RHOL, right heart obstructive lesions; SF, Sylvian fissure; TGA, transposition of the great arteries; ToF, tetralogy of Fallot; US, ultrasound; wks, weeks.

^a^ Axial view.

^b^ Transventricular axial view.

^c^ Transcerebellar coronal view.

### 3.3 Main Findings

The main findings of each study are summarized in Table 3.

**TABLE 3:** Main findings of the studies included.

Author	Intra‐ and interobserver reliability	Cortical development findings	Cortical delay versus Gestational age	MCA Doppler evaluation	Influence of type of CHD on cortical evaluation
Peng et al. (2016)	Not evaluated	SF, POF, and CF values were decreased in CHD fetuses compared to controls (*p* < 0.01) in the second and third scans	Increased delay after 26 weeks compared to 21–25 weeks	Not evaluated	LHOLs were associated with smaller brain fissure depths (SF, POF, and CF, *p* < 0.001)
Koning et al. (2017)	Yes, good reliability	Trajectories of the left insula and right POF were significantly decreased between 22 and 32 weeks in CHD compared to controls (*p* = 0.028 and *p* = 0.043)	Constant delay between different GA evaluated	No significant associations found between CHD and MCA Doppler	Not evaluated
Everwijn et al. (2020)	Not evaluated	Cases had less mature brains than controls at the median GA (26.16 weeks): −3.2 days, 95% CI (1.6; 4.8), *p* < 0.001	Constant delay between different GA evaluated	Not evaluated	TGA and AVSD/ToF fetuses had the less mature brains: −4.0 days, 95% CI (−6.7; −1.2), *p =* 0.006 and −4.5 days, 95% CI (−6.8; −2.3), *p* < 0.001, respectively
He et al. (2017)	Yes, good reliability	The depths of the POF, SF and CF in the ToF group smaller than the control group (*p* < 0.05), except for the depth of SF at 27–28 weeks (*p* = 0.054)	There was no comparison between findings in different GA	Lower MCA PI of ToF fetuses compared to controls (*p* = 0.001)	Not evaluated, only fetuses with ToF included
Rizzo et al. (2022)	Yes, good reliability	Depths of SF and POF reduced in groups A and B compared to controls and among each other (*p* < 0.05). Insula depth increased in groups A and B compared to controls and among each other (*p* < 0.05)	Single evaluation	No significant associations found between CHD and MCA Doppler	Depths of SF and POF were more reduced in group B compared to group A, and the insula depth was more increased in group B compared to group A

Abbreviations: AVSD, atrioventricular septal defect; CF, calcarine fissure; CHD, congenital heart disease; CI, confidence interval; GA, gestational age; LHOL, left heart obstructive lesions; MCA, middle cerebral artery; PI, pulsatility index; POF, parieto‐occipital fissure; SF, Sylvian fissure; TGA, transposition of great arteries; ToF, Tetralogy of Fallot; wks, weeks.

#### 3.3.1 Extent of Cortical Delay

Using a brain‐age prediction algorithm, Everwijn et al. [13] reported a mean delay of 3.2 days in fetuses with heart diseases, mainly transposition of the great arteries (TGA), ToF and atrioventricular septal defect (AVSD), compared to controls. The other studies, using biometric measurements of brain fissure depth, showed statistically significant differences, with the absolute values varying from 1 to 3 mm [11, 12, 22, 23].^.^

#### 3.3.2 Gestational Age at Which Differences Were Observed

Peng et al. [11], Everwijn et al. [13], and He et al. [22] observed that cortical development delays in fetuses with isolated cardiac diseases were more evident after 26 weeks GA. Koning et al. [12] reported significantly decreased trajectories of the left insula and right parieto‐occipital fissure (POF) between 22 and 32 weeks in the CHD group.

#### 3.3.3 Type of Cardiac Disease Versus Brain Oxygenation

Peng et al. [11], Everwijn et al. [13], and Rizzo et al. [23] showed that fetuses with CHD that lead to reduced blood flow to the brain, such as left heart obstructive lesions (LHOL) and TGA, have greater delays in cortical brain development compared with other types of heart defects.

#### 3.3.4 Middle Cerebral Artery (MCA) Doppler Evaluation Versus Cortical Development

The study carried out by He et al. [22] found an association between hemodynamic redistribution and cortical development delay evaluated by MCA Doppler in fetuses with heart defects, while Koning et al. [12] and Rizzo et al. [23] did not observe this association.

#### 3.3.5 Evaluation of Postnatal Neurodevelopment

Only Peng et al. [11] evaluated neurodevelopment in the medium‐term. They observed that children with cardiac diseases had significantly lower scores on the Psychomotor Development Index (PDI) and the Mental Development Index (MDI) of the Bayley Scales of Infant Development (BSID‐II) than control fetuses (*p <* 0.001) [24]. They found these lower scores to correlate with delays in Sylvian fissure (SF) maturation.

#### 3.3.6 Methodological Quality Assessment

The results of quality assessments are shown in Table 4. Four of the five studies were classified as of good quality by the NOS tool [11, 12, 13, 23], but the study by He et al. [22] could not be evaluated regarding the comparability domain, which impeded the classification of quality.

**TABLE 4:** Quality assessment of the studies included using the Newcastle‒Ottawa Scale (NOS) for cohort studies.

Study	Selection	Comparability	Outcome
Peng et al. (2016)	****	*	***
Koning et al. (2017)	****	**	***
Everwijn et al. (2020)	****	*	***
He et al. (2017)	****	—	**
Rizzo et al. (2022)	****	**	***

*Note:* Good quality: 3 or 4 stars in selection domain AND 1 or 2 stars in comparability domain AND 2 or 3 stars in outcome/exposure domain. Fair quality: 2 stars in selection domain AND 1 or 2 stars in comparability domain AND 2 or 3 stars in outcome/exposure domain. Poor quality: 0 or 1 stars in selection domain OR 0 stars in comparability domain OR 0 or 1 stars in outcome/exposure domain.

#### 3.3.7 Risk of Bias

**FIGURE 2:**
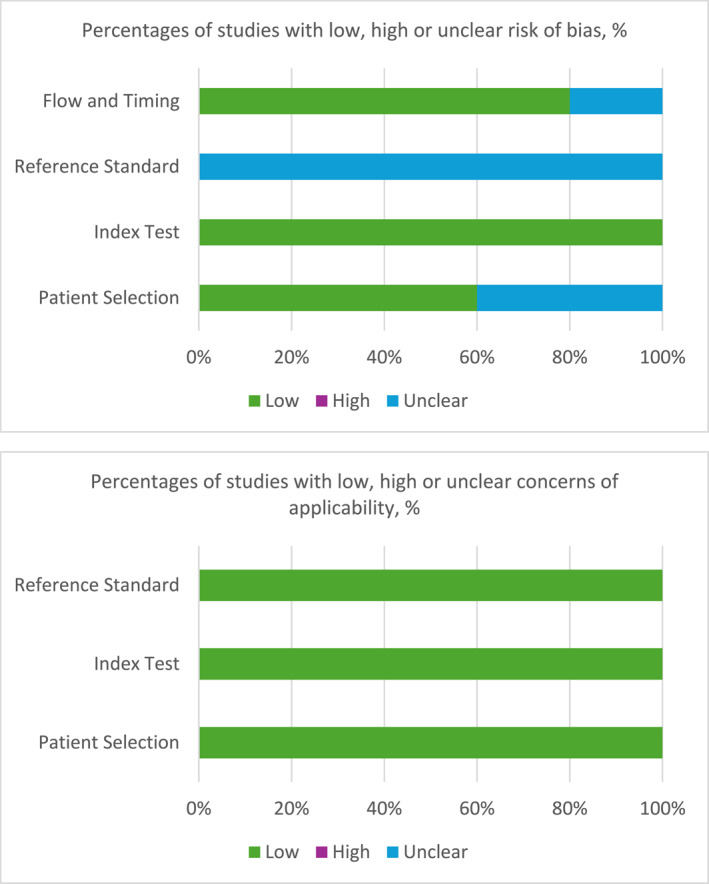
QUADAS‐2 tool results.

The results of the assessment of risk of bias and concerns of applicability using the QUADAS‐2 tool are summarized in Table 5 and in Figure 2. Bias risk was uncertain in two studies because it was unclear whether the patients were consecutively or randomly selected [11, 22]. One of these two studies was also classified as uncertain due to failure to mention the blinding of researchers collecting the measurements [22]. None of the studies provided enough information regarding a standard reference test, giving them an uncertain risk of bias in this regard. Therefore, all studies presented a risk of bias on at least one item. There were no concerns regarding applicability.

**TABLE 5:** QUADAS‐2 tool results.

Study	Risk of bias				Applicability		
	Patient selection	Index test	Reference standard	Flow and timing	Patient selection	Index test	Reference standard
Peng et al. (2016)	?	✓	?	✓	✓	✓	✓
Koning et al. (2017)	✓	✓	?	✓	✓	✓	✓
Everwijn et al. (2020)	✓	✓	?	✓	✓	✓	✓
He et al. (2017)	?	?	?	?	✓	✓	✓
Rizzo et al. (2022)	✓	✓	?	✓	✓	✓	✓

*Note:* ✓, low risk; ✗, high risk; ?, unclear.

## 4 Discussion

This is the first systematic review to investigate ultrasonographic evaluation of brain cortical development in fetuses with cardiac diseases. Taken together, the analyzed studies indicate that ultrasound can detect cortical development delays in fetuses with isolated cardiac abnormalities. These delays are most apparent in the third trimester but are sometimes apparent late in the second trimester.

The first evidence of cortical development delays in fetuses with heart diseases came from MRI studies showing progressive declines in cortical development and volumetric growth of white matter and cortical and subcortical gray matter, as well as reduced brain volume [9, 10, 25, 26]. Neurodevelopmental compromise is the most common morbidity in CHD. It is present in up to 50% of children with moderate to severe CHD [27]. In fetuses with cardiac defects, 20%–30% have some form of CNS abnormality [28]. CNS pathophysiology in CHD fetuses results from a complex interaction of risk factors in each child, including (epi)genetics, prenatal hemodynamic changes, placental functioning, and environmental influences, such as surgical interventions and perioperative care. It is then further influenced by other factors during infancy [27, 29].

Among the studies included in this review, there was a good fissure measurement success rate and good reproducibility, with low intra‐ and interobserver variability. These findings are aligned with those of another study using a similar methodology to evaluate fetal cortical development [30].

Everwijn et al. [13] found a mean cortical development delay of 3.2 days in fetuses with heart diseases compared to controls. Using a grading system proposed by Pistorius et al. [31], another study by Everwijn et al. (not included in this study) found small differences in three of nine cortical areas (Sylvian and cingulate fissures) when comparing fetal brain development between controls and fetuses with CHD [32]. The other studies included in this review showed statistically significant differences, but the absolute values varied and were often modest (e.g., 1–3 mm). These are small numerical differences that may still reflect meaningful trends in fetal neurodevelopment, but their clinical impact remains uncertain, especially without consistent postnatal follow‐up data. Therefore, the interpretation of these findings requires caution, emphasizing the need for further research to determine the clinical relevance of these findings. The extent of the cortical delays found in MRI studies is much greater than in ultrasound studies, suggesting that MRI could be a more precise or even a more sensible method [9, 10, 33].

Peng et al. [11], Everwijn et al. [13], and He et al. [22] observed that cortical development delays in fetuses with isolated cardiac diseases are more evident after 26 weeks GA. This is in accordance with MRI studies [10, 33]. Koning et al. [12] and Everwijn et al. [13] found that fetal cardiac defects are associated with regionally delayed cortical development trajectories but also accelerated brain fissure growth, which could be compensatory. During the third trimester, the volume of the fetal cortex increases fourfold due to the peak of neuronal migration, dendritic expansion, synaptogenesis, and glial proliferation [34]. A low supply of substrate likely leads to dysmaturation and inadequate brain growth.

Peng et al. [11], Everwijn et al. [13], and Rizzo et al. [23] showed that low brain oxygenation correlates with greater delays in cortical brain development among fetuses with heart diseases classically associated with reduced blood flow to the brain. This is consistent with previous studies of cortical and subcortical development and brain volume in fetuses with heart defects [26, 35, 36]. However, Masoller et al. [9] found no association between the type of CHD and different degrees of abnormal brain development. Other publications have reported abnormal neurodevelopment in infants with cardiac problems who showed no prenatal alterations in brain perfusion [2, 37]. Therefore, it seems that cardiac disease type alone is not sufficient to predict abnormal brain development or its degree.

Only He et al. [22] found an association between hemodynamic redistribution and cortical development delay in fetuses with heart defects; Koning et al. [12] and Rizzo et al. [23] did not observe this association. The literature is very divided on this matter. While Ruiz et al. [38] did not find an association, Masoller et al. [9] evidenced lower pulsatility index (PI) and cerebroplacental ratio (CPR) in CHD fetuses. In a systematic review, Khalil et al. [28] observed significantly lower CPR in fetuses with cardiac defects than in controls in more than half of the studies reviewed (8 out of 14), while the MCA Doppler was significantly altered in CHD fetuses compared to controls in most of the studies included (10 out of 14).

MCA Doppler and CPR interpretations seem to be more complex in CHD than in normal fetuses. The arteries of the brain have an autoregulatory system that can cause a vasodilatory response to low oxygen and glucose levels [39]. Fetuses with heart defects may present with more pronounced brain artery dilatation in the third and late second trimesters. This is thought to be an adaptive mechanism to increase blood flow, and so oxygen and metabolites, to the brain, as evidenced by low MCA PI [40].

The relationship between brain vasodilation and growth is not fully understood. Reduced brain volume is common in fetuses with CHD, even those with low PI [35]. This suggests that the brain‐sparing effects of low PI are insufficient to guarantee normal brain growth. Prolonged periods of hypoxia may provoke a tendency to normalize brain vascular tonus and consequently diminish neuronal and glial metabolism. This, in turn, may reduce brain oxygen consumption and development [35, 40].

Peng et al. [24] observed that fetuses with cardiac diseases had significantly lower scores on BSID‐II than control fetuses (*p <* 0.001). They found these lower scores to correlate with delays in SF maturation. This is in accordance with other studies that have shown associations between smaller brain volume and sulcation delays with maturation and long‐term neurological outcomes [9, 41].

### 4.1 Quality, Strengths, and Weaknesses

The studies were classified as having good methodological quality overall, but all presented some risk of bias. The main issue was the lack of comparison of the ultrasound findings with MRI, which is currently the gold standard in the evaluation of fetal cortical development. Furthermore, none of the studies performed postnatal evaluations, either by ultrasound or MRI, and four of the five studies did not perform any postnatal neurological evaluation.

The main strength of this review was the use of a predefined protocol with a stringent methodology. Its weaknesses were the small number of articles, and the heterogeneity of the studies included, which prevented a meta‐analysis and limited the conclusions regarding the real‐life impact of the cortical delay in the children's neurodevelopment.

### 4.2 Practical Implications

There is increasing evidence that CHD has harmful effects on fetal brain development. A deeper understanding of this relationship may help identify fetuses at greater risk of neurodevelopmental delay.

Despite the methodological heterogeneity of the studies reviewed, we believe that they demonstrate the utility of fetal ultrasound in the evaluation of fetal brain development. The approach is accessible and low cost when using existing ultrasound facilities and allows for serial evaluations during pregnancy. Nonetheless, targeted neurosonography is a relatively recent tool and should be further explored in larger trials to help establish a uniform assessment methodology and demonstrate its ability to detect clinically relevant delays.

## 5 Conclusions

Fetal cortical development can be successfully assessed using targeted neurosonography or specialized ultrasound. Fetuses with isolated cardiac diseases present with some degree of delayed cortical development, but long‐term postnatal studies are needed to assess if it correlates with impaired neurodevelopment. Identifying such delays *in utero* can optimize the effects of preventive and treatment measures and provide time for parent counseling.

## Ethics Statement

The authors have nothing to report.

## Consent

The authors have nothing to report.

## Conflicts of Interest

The authors declare no conflicts of interest.

## Data Availability

Data sharing not applicable to this article as no datasets were generated or analyzed during the current study.

## Appendix A Search Strategy According to Electronic Databases.

**Table:**

Database	PubMed	Date of search	01/22/2024
#1	((((((((((((((heart defect, congenital [MeSH terms]) OR (heart defects, congenital [MeSH terms])) OR (abnormalities, heart [MeSH terms])) OR (abnormality, heart [MeSH terms])) OR (congenital heart defect [MeSH terms])) OR (congenital heart defects [MeSH terms])) OR (defect, congenital heart [MeSH terms])) OR (defects, congenital heart [MeSH terms])) OR (disease, congenital heart [Other Term])) OR (heart abnormalities [MeSH terms])) OR (heart abnormality [MeSH terms])) OR (heart defect, congenital [MeSH terms])) OR (heart disease, congenital [Other Term])) OR (heart, malformation of [Other Term])) OR (malformation of heart [Other Term])		
#2	((((((((((((((((((((((((cerebral cortical dysplasia [MeSH terms]) OR (cerebral cortical dysplasias [MeSH terms])) OR (cerebral cortical development)) OR (cerebral cortical maturation)) OR (cerebral cortical folding)) OR (cortical development malformation [MeSH terms])) OR (cortical development malformations [MeSH terms])) OR (cortical dysplasia [MeSH terms])) OR (cortical dysplasia, cerebral [MeSH terms])) OR (cortical dysplasia, generalized [Other Term])) OR (cortical dysplasias [MeSH terms])) OR (cortical dysplasias, cerebral [MeSH terms])) OR (development malformation, cortical [MeSH terms])) OR (development malformations, cortical [MeSH terms])) OR (dysplasia, cerebral cortical [MeSH terms])) OR (dysplasia, cortical [MeSH terms])) OR (dysplasia, generalized cortical [Other Term])) OR (generalized cortical dysplasia [Other Term])) OR (generalized cortical dysplasias [Other Term])) OR (dysplasias, cerebral cortical [MeSH terms])) OR (dysplasias, cortical [MeSH terms])) OR (malformations of cerebral cortex development [MeSH terms])) OR (cortex gyration)) OR (cortical maturation)) OR (cortical folding)		
#3	((((((((((((((((((((((((((((((((diagnoses, prenatal ultrasound) OR (diagnoses, ultrasonographic prenatal)) OR (diagnosis, prenatal ultrasound)) OR (diagnosis, ultrasonographic prenatal)) OR (fetal ultrasound)) OR (prenatal diagnoses, ultrasound)) OR (prenatal diagnosis, ultrasound)) OR (prenatal ultrasonographic diagnoses)) OR (prenatal ultrasonographic diagnosis)) OR (prenatal ultrasound)) OR (ultrasonographic diagnoses, prenatal)) OR (ultrasonographic diagnosis, prenatal)) OR (ultrasonographic prenatal diagnoses)) OR (ultrasonographic prenatal diagnosis)) OR (neurosonography)) OR (fetal scan)) OR (prenatal scan)) OR (obstetric scan)) OR (obstetric scans)) OR (ultrasonography, fetal [MeSH terms])) OR (ultrasonic prenatal diagnosis [MeSH terms])) OR (ultrasonic prenatal diagnoses [MeSH terms])) OR (ultrasonic diagnoses, prenatal [MeSH terms])) OR (ultrasonic diagnosis, prenatal [MeSH terms])) OR (prenatal ultrasonography [MeSH terms])) OR (prenatal ultrasonic diagnosis [MeSH terms])) OR (prenatal ultrasonic diagnoses [MeSH terms])) OR (prenatal diagnosis, ultrasonic [MeSH terms])) OR (prenatal diagnoses, ultrasonic [MeSH terms])) OR (fetal ultrasonography [MeSH terms])) OR (diagnosis, ultrasonic prenatal [MeSH terms])) OR (diagnoses, ultrasonic prenatal [MeSH terms])) OR (diagnoses, prenatal ultrasonic [MeSH terms])		
#4	#1 AND #2 AND #3		
Number of identified citations			45

Database	Cochrane library	Date of search	01/24/2024
#1	(heart defect, congenital) OR (heart defects, congenital) OR (abnormalities, heart) OR (abnormality, heart) OR (congenital heart defect) OR (congenital heart defects) OR (defect, congenital heart) OR (defects, congenital heart) OR (disease, congenital heart) OR (heart abnormalities) OR (heart abnormality) OR (heart defect, congenital) OR (heart disease, congenital) OR (heart, malformation of) OR (malformation of heart)		
#2	(cerebral cortical dysplasia) OR (cerebral cortical dysplasias) OR (cerebral cortical development) OR (cerebral cortical maturation) OR (cerebral cortical folding) OR (cortical development malformation) OR (cortical development malformations) OR (cortical dysplasia) OR (cortical dysplasia, cerebral) OR (cortical dysplasia, generalized) OR (cortical dysplasias) OR (cortical dysplasias, cerebral) OR (development malformation, cortical) OR (development malformations, cortical) OR (dysplasia, cerebral cortical) OR (dysplasia, cortical) OR (dysplasia, generalized cortical) OR (generalized cortical dysplasia) OR (generalized cortical dysplasias) OR (dysplasias, cerebral cortical) OR (dysplasias, cortical) OR (malformations of cerebral cortex development) OR (cortex gyration) OR (cortical maturation) OR (cortical folding)		
#3	(diagnoses, prenatal ultrasound) OR (diagnoses, ultrasonographic prenatal) OR (diagnosis, prenatal ultrasound) OR (diagnosis, ultrasonographic prenatal) OR (fetal ultrasound) OR (prenatal diagnoses, ultrasound) OR (prenatal diagnosis, ultrasound) OR (prenatal ultrasonographic diagnoses) OR (prenatal ultrasonographic diagnosis) OR (prenatal ultrasound) OR (ultrasonographic diagnoses, prenatal) OR (ultrasonographic diagnosis, prenatal) OR (ultrasonographic prenatal diagnoses) OR (ultrasonographic prenatal diagnosis) OR (neurosonography) OR (fetal scan) OR (prenatal scan) OR (obstetric scan) OR (obstetric scans) OR (ultrasonography, fetal) OR (ultrasonic prenatal diagnosis) OR (ultrasonic prenatal diagnoses) OR (ultrasonic diagnoses, prenatal) OR (ultrasonic diagnosis, prenatal) OR (prenatal ultrasonography) OR (prenatal ultrasonic diagnosis) OR (prenatal ultrasonic diagnoses) OR (prenatal diagnosis, ultrasonic) OR (prenatal diagnoses, ultrasonic) OR (fetal ultrasonography) OR (diagnosis, ultrasonic prenatal) OR (diagnoses, ultrasonic prenatal) OR (diagnoses, prenatal ultrasonic)		
#4	#1 AND #2 AND #3		
Number of citations identified			10

Database	Biblioteca Virtual em Saúde	Date of search	23/01/2024
#1	((heart defect, congenital) OR (heart defects, congenital) OR (abnormalities, heart) OR (abnormality, heart) OR (congenital heart defect) OR (congenital heart defects) OR (defect, congenital heart) OR (defects, congenital heart) OR (disease, congenital heart) OR (heart abnormalities) OR (heart abnormality) OR (heart defect, congenital) OR (heart disease, congenital) OR (heart, malformation of) OR (malformation of heart))		
#2	((cerebral cortical dysplasia) OR (cerebral cortical dysplasias) OR (cerebral cortical development) OR (cerebral cortical maturation) OR (cerebral cortical folding) OR (cortical development malformation) OR (cortical development malformations) OR (cortical dysplasia) OR (cortical dysplasia, cerebral) OR (cortical dysplasia, generalized) OR (cortical dysplasias) OR (cortical dysplasias, cerebral) OR (development malformation, cortical) OR (development malformations, cortical) OR (dysplasia, cerebral cortical) OR (dysplasia, cortical) OR (dysplasia, generalized cortical) OR (generalized cortical dysplasia) OR (generalized cortical dysplasias) OR (dysplasias, cerebral cortical) OR (dysplasias, cortical) OR (malformations of cerebral cortex development) OR (cortex gyration) OR (cortical maturation) OR (cortical folding))		
#3	((diagnoses, prenatal ultrasound) OR (diagnoses, ultrasonographic prenatal) OR (diagnosis, prenatal ultrasound) OR (diagnosis, ultrasonographic prenatal) OR (fetal ultrasound) OR (prenatal diagnoses, ultrasound) OR (prenatal diagnosis, ultrasound) OR (prenatal ultrasonographic diagnoses) OR (prenatal ultrasonographic diagnosis) OR (prenatal ultrasound) OR (ultrasonographic diagnoses, prenatal) OR (ultrasonographic diagnosis, prenatal) OR (ultrasonographic prenatal diagnoses) OR (ultrasonographic prenatal diagnosis) OR (neurosonography) OR (fetal scan) OR (prenatal scan) OR (obstetric scan) OR (obstetric scans) OR (ultrasonography, fetal) OR (ultrasonic prenatal diagnosis) OR (ultrasonic prenatal diagnoses) OR (ultrasonic diagnoses, prenatal) OR (ultrasonic diagnosis, prenatal) OR (prenatal ultrasonography) OR (prenatal ultrasonic diagnosis) OR (prenatal ultrasonic diagnoses) OR (prenatal diagnosis, ultrasonic) OR (prenatal diagnoses, ultrasonic) OR (fetal ultrasonography) OR (diagnosis, ultrasonic prenatal) OR (diagnoses, ultrasonic prenatal) OR (diagnoses, prenatal ultrasonic))		
#4	#1 AND #2 AND #3		
Number of citations identified			67

Database	Epistemonikos	Date of search	23/01/2024
#1	(cerebral cortical dysplasia) OR (cerebral cortical dysplasias) OR (cerebral cortical development) OR (cerebral cortical maturation) OR (cerebral cortical folding) OR (cortical development malformation) OR (cortical development malformations) OR (cortical dysplasia) OR (cortical dysplasia, cerebral) OR (cortical dysplasia, generalized) OR (cortical dysplasias) OR (cortical dysplasias, cerebral) OR (development malformation, cortical) OR (development malformations, cortical) OR (dysplasia, cerebral cortical) OR (dysplasia, cortical) OR (dysplasia, generalized cortical) OR (generalized cortical dysplasia) OR (generalized cortical dysplasias) OR (dysplasias, cerebral cortical) OR (dysplasias, cortical) OR (malformations of cerebral cortex development) OR (cortex gyration) OR (cortical maturation) OR (cortical folding)		
#2	(diagnoses, prenatal ultrasound) OR (diagnoses, ultrasonographic prenatal) OR (diagnosis, prenatal ultrasound) OR (diagnosis, ultrasonographic prenatal) OR (fetal ultrasound) OR (prenatal diagnoses, ultrasound) OR (prenatal diagnosis, ultrasound) OR (prenatal ultrasonographic diagnoses) OR (prenatal ultrasonographic diagnosis) OR (prenatal ultrasound) OR (ultrasonographic diagnoses, prenatal) OR (ultrasonographic diagnosis, prenatal) OR (ultrasonographic prenatal diagnoses) OR (ultrasonographic prenatal diagnosis) OR (neurosonography) OR (fetal scan) OR (prenatal scan) OR (obstetric scan) OR (obstetric scans) OR (ultrasonography, fetal) OR (ultrasonic prenatal diagnosis) OR (ultrasonic prenatal diagnoses) OR (ultrasonic diagnoses, prenatal) OR (ultrasonic diagnosis, prenatal) OR (prenatal ultrasonography) OR (prenatal ultrasonic diagnosis) OR (prenatal ultrasonic diagnoses) OR (prenatal diagnosis, ultrasonic) OR (prenatal diagnoses, ultrasonic) OR (fetal ultrasonography) OR (diagnosis, ultrasonic prenatal) OR (diagnoses, ultrasonic prenatal) OR (diagnoses, prenatal ultrasonic)		
#3	(title:((heart defect, congenital) OR (heart defects, congenital) OR (abnormalities, heart) OR (abnormality, heart) OR (congenital heart defect) OR (congenital heart defects) OR (defect, congenital heart) OR (defects, congenital heart) OR (disease, congenital heart) OR (heart abnormalities) OR (heart abnormality) OR (heart defect, congenital) OR (heart disease, congenital) OR (heart, malformation of) OR (malformation of heart)) OR abstract:((heart defect, congenital) OR (heart defects, congenital) OR (abnormalities, heart) OR (abnormality, heart) OR (congenital heart defect) OR (congenital heart defects) OR (defect, congenital heart) OR (defects, congenital heart) OR (disease, congenital heart) OR (heart abnormalities) OR (heart abnormality) OR (heart defect, congenital) OR (heart disease, congenital) OR (heart, malformation of) OR (malformation of heart))) AND (title:((cerebral cortical dysplasia) OR (cerebral cortical dysplasias) OR (cerebral cortical development) OR (cerebral cortical maturation) OR (cerebral cortical folding) OR (cortical development malformation) OR (cortical development malformations) OR (cortical dysplasia) OR (cortical dysplasia, cerebral) OR (cortical dysplasia, generalized) OR (cortical dysplasias) OR (cortical dysplasias, cerebral) OR (development malformation, cortical) OR (development malformations, cortical) OR (dysplasia, cerebral cortical) OR (dysplasia, cortical) OR (dysplasia, generalized cortical) OR (generalized cortical dysplasia) OR (generalized cortical dysplasias) OR (dysplasias, cerebral cortical) OR (dysplasias, cortical) OR (malformations of cerebral cortex development) OR (cortex gyration) OR (cortical maturation) OR (cortical folding)) OR abstract:((cerebral cortical dysplasia) OR (cerebral cortical dysplasias) OR (cerebral cortical development) OR (cerebral cortical maturation) OR (cerebral cortical folding) OR (cortical development malformation) OR (cortical development malformations) OR (cortical dysplasia) OR (cortical dysplasia, cerebral) OR (cortical dysplasia, generalized) OR (cortical dysplasias) OR (cortical dysplasias, cerebral) OR (development malformation, cortical) OR (development malformations, cortical) OR (dysplasia, cerebral cortical) OR (dysplasia, cortical) OR (dysplasia, generalized cortical) OR (generalized cortical dysplasia) OR (generalized cortical dysplasias) OR (dysplasias, cerebral cortical) OR (dysplasias, cortical) OR (malformations of cerebral cortex development) OR (cortex gyration) OR (cortical maturation) OR (cortical folding))) AND (title:((diagnoses, prenatal ultrasound) OR (diagnoses, ultrasonographic prenatal) OR (diagnosis, prenatal ultrasound) OR (diagnosis, ultrasonographic prenatal) OR (fetal ultrasound) OR (prenatal diagnoses, ultrasound) OR (prenatal diagnosis, ultrasound) OR (prenatal ultrasonographic diagnoses) OR (prenatal ultrasonographic diagnosis) OR (prenatal ultrasound) OR (ultrasonographic diagnoses, prenatal) OR (ultrasonographic diagnosis, prenatal) OR (ultrasonographic prenatal diagnoses) OR (ultrasonographic prenatal diagnosis) OR (neurosonography) OR (fetal scan) OR (prenatal scan) OR (obstetric scan) OR (obstetric scans) OR (ultrasonography, fetal) OR (ultrasonic prenatal diagnosis) OR (ultrasonic prenatal diagnoses) OR (ultrasonic diagnoses, prenatal) OR (ultrasonic diagnosis, prenatal) OR (prenatal ultrasonography) OR (prenatal ultrasonic diagnosis) OR (prenatal ultrasonic diagnoses) OR (prenatal diagnosis, ultrasonic) OR (prenatal diagnoses, ultrasonic) OR (fetal ultrasonography) OR (diagnosis, ultrasonic prenatal) OR (diagnoses, ultrasonic prenatal) OR (diagnoses, prenatal ultrasonic)) OR abstract:((diagnoses, prenatal ultrasound) OR (diagnoses, ultrasonographic prenatal) OR (diagnosis, prenatal ultrasound) OR (diagnosis, ultrasonographic prenatal) OR (fetal ultrasound) OR (prenatal diagnoses, ultrasound) OR (prenatal diagnosis, ultrasound) OR (prenatal ultrasonographic diagnoses) OR (prenatal ultrasonographic diagnosis) OR (prenatal ultrasound) OR (ultrasonographic diagnoses, prenatal) OR (ultrasonographic diagnosis, prenatal) OR (ultrasonographic prenatal diagnoses) OR (ultrasonographic prenatal diagnosis) OR (neurosonography) OR (fetal scan) OR (prenatal scan) OR (obstetric scan) OR (obstetric scans) OR (ultrasonography, fetal) OR (ultrasonic prenatal diagnosis) OR (ultrasonic prenatal diagnoses) OR (ultrasonic diagnoses, prenatal) OR (ultrasonic diagnosis, prenatal) OR (prenatal ultrasonography) OR (prenatal ultrasonic diagnosis) OR (prenatal ultrasonic diagnoses) OR (prenatal diagnosis, ultrasonic) OR (prenatal diagnoses, ultrasonic) OR (fetal ultrasonography) OR (diagnosis, ultrasonic prenatal) OR (diagnoses, ultrasonic prenatal) OR (diagnoses, prenatal ultrasonic)))		
Number of citations identified		13	

*Source:* written by the authors.

## References

[1] B. Khoshnood , N. Lelong , L. Houyel , et al., “Prevalence, Timing of Diagnosis and Mortality of Newborns With Congenital Heart Defects: A Population‐Based Study,” Heart 98, no. 22 (November 15, 2012): 1667–1673, 10.1136/heartjnl-2012-302543. 22888161

[2] A. Majnemer , C. Limperopoulos , M. I. Shevell , C. Rohlicek , B. Rosenblatt , and C. Tchervenkov , “A New Look at Outcomes of Infants With Congenital Heart Disease,” Pediatric Neurology 40, no. 3 (March 2009): 197–204, 10.1016/j.pediatrneurol.2008.09.014. 19218033

[3] J. I. E. Hoffman and S. Kaplan , “The Incidence of Congenital Heart Disease,” Journal of the American College of Cardiology 39, no. 12 (June 2002): 1890–1900, 10.1016/s0735-1097(02)01886-7. 12084585

[4] R. L. Knowles , C. Bull , C. Wren , and C. Dezateux , “Mortality With Congenital Heart Defects in England and Wales, 1959–2009: Exploring Technological Change Through Period and Birth Cohort Analysis,” Archives of Disease in Childhood 97, no. 10 (October 2012): 861–865, 10.1136/archdischild-2012-301662. 22753769

[5] G. Wernovsky , “Outcomes Regarding the Central Nervous System in Children With Complex Congenital Cardiac Malformations,” supplement, Cardiology in the Young 15, no. S1 (February 8, 2005): 132–133, 10.1017/s1047951105001162. 15934705

[6] M. T. Donofrio and A. N. Massaro , “Impact of Congenital Heart Disease on Brain Development and Neurodevelopmental Outcome,” International Journal of Pediatrics 2010 (2010): 1–13, 10.1155/2010/359390. PMC2938447 20862365

[7] D. J. Licht , D. M. Shera , R. R. Clancy , et al., “Brain Maturation Is Delayed in Infants With Complex Congenital Heart Defects,” Journal of Thoracic and Cardiovascular Surgery 137, no. 3 (March 2009): 529–537, 10.1016/j.jtcvs.2008.10.025. 19258059 PMC2701902

[8] F. A. R. Jansen , S. M. P. Everwijn , R. Scheepjens , et al., “Fetal Brain Imaging in Isolated Congenital Heart Defects – a Systematic Review and Meta‐Analysis,” Prenatal Diagnosis 36, no. 7 (July 19, 2016): 601–613, 10.1002/pd.4842. 27187181

[9] N. Masoller , M. Sanz‐Cortés , F. Crispi , et al., “Mid‐Gestation Brain Doppler and Head Biometry in Fetuses With Congenital Heart Disease Predict Abnormal Brain Development at Birth,” Ultrasound in Obstetrics and Gynecology 47, no. 1 (January 5, 2016): 65–73, 10.1002/uog.14919. 26053596

[10] C. Clouchoux , A. J. du Plessis , M. Bouyssi‐Kobar , et al., “Delayed Cortical Development in Fetuses With Complex Congenital Heart Disease,” Cerebral Cortex 23, no. 12 (December 1, 2013): 2932–2943, 10.1093/cercor/bhs281. 22977063

[11] Q. Peng , Q. Zhou , M. Zang , et al., “Reduced Fetal Brain Fissures Depth in Fetuses With Congenital Heart Diseases,” Prenatal Diagnosis 36, no. 11 (November 1, 2016): 1047–1053, 10.1002/pd.4931. 27681656

[12] I. V. Koning , A. W. van Graafeiland , I. A. L. Groenenberg , et al., “Prenatal Influence of Congenital Heart Defects on Trajectories of Cortical Folding of the Fetal Brain Using Three‐Dimensional Ultrasound,” Prenatal Diagnosis 37, no. 10 (October 1, 2017): 1008–1016, 10.1002/pd.5135. 28768058

[13] S. M. P. Everwijn , A. I. L. Namburete , N. van Geloven , et al., “The Association Between Flow and Oxygenation and Cortical Development in Fetuses With Congenital Heart Defects Using a Brain‐Age Prediction Algorithm,” Prenatal Diagnosis 41, no. 1 (January 1, 2021): 43–51, 10.1002/pd.5813. 33448406 PMC7891604

[14] G. Malinger , D. Paladini , K. K. Haratz , A. Monteagudo , G. L. Pilu , and I. E. Timor‐Tritsch , “ISUOG Practice Guidelines (Updated): Sonographic Examination of the Fetal Central Nervous System. Part 1: Performance of Screening Examination and Indications for Targeted Neurosonography,” Ultrasound in Obstetrics and Gynecology 56, no. 3 (September 2020): 476–484, 10.1002/uog.22145. 32870591

[15] A. Sotiriadis , S. I. Papatheodorou , and W. P. Martins , “Synthesizing Evidence From Diagnostic Accuracy TEsts: The SEDATE Guideline,” Ultrasound in Obstetrics and Gynecology 47, no. 3 (March 10, 2016): 386–395, 10.1002/uog.15762. 26411461

[16] M. J. Page , J. E. McKenzie , P. M. Bossuyt , et al., “The PRISMA 2020 Statement: An Updated Guideline for Reporting Systematic Reviews,” BMJ 372 (2021): n71, 10.1136/bmj.n71. 33782057 PMC8005924

[17] “Systematic Reviews, Step by Step Constructing a Search Strategy and Searching for Evidence,” [Internet], http://journals.lww.com/ajnonline.

[18] [Internet]. PubReMiner, http://hgserver2.amc.nl/cgi‐bin/miner/miner2.cgi.

[19] M. Ouzzani , H. Hammady , Z. Fedorowicz , and A. Elmagarmid , “Rayyan‐A Web and Mobile App for Systematic Reviews,” Systematic Reviews 5, no. 1 (December 5, 2016): 210, 10.1186/s13643-016-0384-4. 27919275 PMC5139140

[20] G. Wells , B. Shea , D. O’ Connell , et al., “The Newcastle‐Ottawa Scale (NOS) for Assessing the Quality of Nonrandomised Studies in Meta‐Analyses,” [Internet]. [cited 2024 Mar 11], https://www.ohri.ca/programs/clinical_epidemiology/oxford.asp.

[21] P. F. Whiting , A. W. Rutjes , M. E. Westwood , et al., “QUADAS‐2: A Revised Tool for the Quality Assessment of Diagnostic Accuracy Studies,” Annals of Internal Medicine 155, no. 8 (October 18, 2011): 529–536, 10.7326/0003-4819-155-8-201110180-00009. 22007046

[22] J. He , S. Li , C. Xi , W. Huaxuan , Y. Yuan , and L. Dandan , “A Prenatal Ultrasound Study of Cerebral Sulci and Gyrus Development in Fetuses With Tetralogy of Fallot,” Journal of Southern Medical University [Internet] 37, no. 6 (2017): 721–729, http://www.j‐smu.com. 28669943 10.3969/j.issn.1673-4254.2017.06.02 PMC6744134

[23] G. Rizzo , M. E. Pietrolucci , M. De Vito , M. Pavjola , A. Capponi , and I. Mappa , “Fetal Brain Biometry and Cortical Development in Congenital Heart Disease: A Prospective Cross Sectional Study,” Journal of Clinical Ultrasound 51, no. 1 (January 1, 2023): 84–90, 10.1002/jcu.23308. 36069371

[24] N. Bayley , Manual for the Bayley Scales of Infant Development, 2nd ed. (Psychological Corporation, 1993).

[25] C. Limperopoulos , W. Tworetzky , D. B. McElhinney , et al., “Brain Volume and Metabolism in Fetuses With Congenital Heart Disease,” Circulation 121, no. 1 (January 5, 2010): 26–33, 10.1161/circulationaha.109.865568. 20026783 PMC2819908

[26] M. H. Lauridsen , N. Uldbjerg , O. B. Petersen , et al., “Fetal Heart Defects and Measures of Cerebral Size,” Journal of Pediatrics 210 (July 2019): 146–153, 10.1016/j.jpeds.2019.02.042. 30961987

[27] G. Wernovsky and D. J. Licht , “Neurodevelopmental Outcomes in Children With Congenital Heart Disease—What Can We Impact?,” Pediatric Critical Care Medicine 17, no. 8 (August 2016): S232–S242, 10.1097/pcc.0000000000000800. 27490605 PMC4975480

[28] A. Khalil , S. Bennet , B. Thilaganathan , D. Paladini , P. Griffiths , and J. S. Carvalho , “Prevalence of Prenatal Brain Abnormalities in Fetuses With Congenital Heart Disease: A Systematic Review,” Ultrasound in Obstetrics and Gynecology 48, no. 3 (September 2016): 296–307, 10.1002/uog.15932. 27062519

[29] J. Rychik , D. Goff , E. McKay , et al., “Characterization of the Placenta in the Newborn With Congenital Heart Disease: Distinctions Based on Type of Cardiac Malformation,” Pediatric Cardiology 39, no. 6 (August 4, 2018): 1165–1171, 10.1007/s00246-018-1876-x. 29728721 PMC6096845

[30] N. Hahner , B. Puerto , M. Perez‐Cruz , et al., “Altered Cortical Development in Fetuses With Isolated Nonsevere Ventriculomegaly Assessed by Neurosonography,” Prenatal Diagnosis 38, no. 5 (April 19, 2018): 365–375, 10.1002/pd.5240. 29458235

[31] L. R. Pistorius , P. Stoutenbeek , F. Groenendaal , et al., “Grade and Symmetry of Normal Fetal Cortical Development: A Longitudinal Two‐ and Three‐Dimensional Ultrasound Study,” Ultrasound in Obstetrics and Gynecology 36, no. 6 (December 24, 2010): 700–708, 10.1002/uog.7705. 20521241

[32] S. M. Everwijn , J. F. van Bohemen , N. van Geloven , et al., “Serial Neurosonography in Fetuses With Congenital Heart Defects Shows Mild Delays in Cortical Development,” Prenatal Diagnosis 41, no. 13 (December 19, 2021): 1649–1657, 10.1002/pd.6038. 34474501 PMC9293037

[33] C. M. Ortinau , C. K. Rollins , A. Gholipour , et al., “Early‐Emerging Sulcal Patterns Are Atypical in Fetuses With Congenital Heart Disease,” Cerebral Cortex 29, no. 8 (July 22, 2019): 3605–3616, 10.1093/cercor/bhy235. 30272144 PMC6644862

[34] C. Ortinau and J. Neil , “The Neuroanatomy of Prematurity: Normal Brain Development and the Impact of Preterm Birth,” Clinical Anatomy 28, no. 2 (March 15, 2015): 168–183, 10.1002/ca.22430. 25043926

[35] L. Sun , C. K. Macgowan , J. G. Sled , et al., “Reduced Fetal Cerebral Oxygen Consumption Is Associated With Smaller Brain Size in Fetuses With Congenital Heart Disease,” Circulation 131, no. 15 (April 14, 2015): 1313–1323, 10.1161/circulationaha.114.013051. 25762062 PMC4398654

[36] M. Pérez‐Cruz , O. Gómez , M. Gibert , et al., “Corpus Callosum Size by Neurosonography in Fetuses With Congenital Heart Defect and Relationship With Expected Pattern of Brain Oxygen Supply,” Ultrasound in Obstetrics and Gynecology 59, no. 2 (February 12, 2022): 220–225, 10.1002/uog.23684. 33998077

[37] M. T. Donofrio , Y. A. Bremer , R. M. Schieken , et al., “Autoregulation of Cerebral Blood Flow in Fetuses With Congenital Heart Disease: The Brain Sparing Effect,” Pediatric Cardiology 24, no. 5 (September 1, 2003): 436–443, 10.1007/s00246-002-0404-0. 14627309

[38] A. Ruiz , M. Cruz‐Lemini , N. Masoller , et al., “Longitudinal Changes in Fetal Biometry and Cerebroplacental Hemodynamics in Fetuses With Congenital Heart Disease,” Ultrasound in Obstetrics and Gynecology 49, no. 3 (March 6, 2017): 379–386, 10.1002/uog.15970. 27214694

[39] D. Oros , F. Figueras , R. Cruz‐Martinez , E. Meler , M. Munmany , and E. Gratacos , “Longitudinal Changes in Uterine, Umbilical and Fetal Cerebral Doppler Indices in Late‐Onset Small‐For‐Gestational Age Fetuses,” Ultrasound in Obstetrics and Gynecology 37, no. 2 (February 24, 2011): 191–195, 10.1002/uog.7738. 20617509

[40] F. T. Lee , M. Seed , L. Sun , and D. Marini , “Fetal Brain Issues in Congenital Heart Disease,” Translational Pediatrics 10, no. 8 (August 2021): 2182–2196, 10.21037/tp-20-224. 34584890 PMC8429876

[41] A. Sadhwani , D. Wypij , V. Rofeberg , et al., “Fetal Brain Volume Predicts Neurodevelopment in Congenital Heart Disease,” Circulation 145, no. 15 (April 12, 2022): 1108–1119, 10.1161/circulationaha.121.056305. 35143287 PMC9007882

